# Testing for Abrasion Resistance of WC-Co Composites for Blades Used in Wood-Based Material Processing

**DOI:** 10.3390/ma16175836

**Published:** 2023-08-25

**Authors:** Joanna Wachowicz, Joanna Fik, Zbigniew Bałaga, Grzegorz Stradomski

**Affiliations:** 1 Department of Mechanical Processing of Wood, Institute of Wood Sciences and Furniture, Warsaw University of Life Sciences, Nowoursynowska Street, 166, 02-787 Warsaw, Poland; 2 Faculty of Science and Technology, Jan Dlugosz University in Czestochowa, Armii Krajowej Street 13/15, 42-200 Czestochowa, Poland; j.fik@ujd.edu.pl; 3 Faculty of Production Engineering and Materials Technology, Czestochowa University of Technology, Armii Krajowej Street, 19, 42-201 Czestochowa, Poland; zbigniew.balaga@pcz.pl (Z.B.); grzegorz.stradomski@pcz.pl (G.S.)

**Keywords:** sintering, cemented carbides, WC-Co, abrasive wear resistance

## Abstract

Commonly used tool materials for machining wood-based materials are WC-Co carbides. Although they have been known for a long time, there is still much development in the field of sintered tool materials, especially WC-Co carbides and superhard materials. The use of new manufacturing methods (such as FAST—field-assisted sintering technology), which use pulses of electric current for heating, can improve the properties of the materials used for cutting tools, thereby increasing the cost-effectiveness of machining. The ability to increase tool life without the downtime associated with tool wear allows significant cost savings, particularly in mass production. This paper presents the results of a study of the effect of grain size and cobalt content of carbide tool sinters on the tribological properties of the materials studied. The powders used for consolidation were characterised by irregular shape and formed agglomerates of different sizes. Tribological tests were carried out using the T-01 (ball-on-disc) method. In order to determine the wear kinetics, the entire friction path was divided into 15 cycles of 200 m and the weight loss was measured after each stage. In order to determine the mechanism and intensity of wear of the tested materials under technically dry friction conditions, the surface of the tested sinters was observed before the test and after 5, 10, and 15 cycles. The conclusions of the study indicate that the predominant effect of surface cooperation at the friction node is abrasion due to the material chipping that occurs during the process. The results confirm the influence of sintered grain size and cobalt content on durability. In the context of the application of the materials in question for cutting tools, it can be pointed out that sintered WC(0.4)_4 has the highest potential for use in the manufacture of cutting tools.

## 1. Introduction

The furniture industry is a crucial sector of the economy. As furniture production increases, technological advancements in the machining machinery field also improve, leading to the need for novel tool materials that meet rising requirements. Wood-based materials, including MDF, HDF, and particleboard, constitute 90% of the materials employed in the furniture industry [1,2,3,4,5,6].

Machining wood-based materials is challenging. These materials have a heterogeneous structure, a complex chemical composition, and anisotropy, which necessitate the use of distinct techniques for machining compared to metals. Furthermore, such materials have low thermal conductivity, leading to a substantial increase in tool wear. Accelerated tool wear is particularly significant when machining particleboards, which have an increased fraction of mineral impurities in comparison to MDF, at high cutting speeds [7,8,9,10,11,12,13,14,15,16].

WC-Co carbides are commonly used as tool materials for machining wood-based materials. Despite their long-known existence, intense development is still taking place in the field of sintered tool materials, especially for WC-Co carbides and superhard materials [17,18,19,20,21,22,23].

New manufacturing techniques, including FAST (field-assisted sintering technology), which uses pulses of electric current for heating, can improve the properties of materials used for cutting tools. This can increase machining profitability. Extending tool life without downtime due to wear and tear can significantly reduce costs. This is especially important in batch production [24,25,26,27,28,29]. Current research on tool materials for machining chipboard necessitates the evaluation of tribological properties. The tribological wear of tools that operate under abrasive conditions usually happens due to the chipping of fragments from the surface layer. This results from the formation and propagation of microcracks [30,31,32,33,34,35]. According to the literature [36,37], the intensity of abrasive wear on WC-Co cemented carbides is dependent on the microstructure, hardness, and operating conditions. One of the primary objectives of users is to acquire high-quality products that serve as a reliable source. Friction should be minimised to achieve this goal [38]. A previous study has demonstrated that WC-Co nanocomposites with 12% Co exhibit roughly double the abrasive wear resistance of conventional composites [39].

This work emphasises the use of the innovative U-Fast sintering technology to obtain materials intended for tools for processing wood-based materials and their tribological properties. Obtaining WC-Co carbides with a fine-grained structure should result in higher resistance to tribological wear, which is important from the point of view of the application of these materials. Currently, tools obtained by innovative sintering technologies are not used in woodworking.

The aim of this research is to determine the tribological properties of WC-Co sintered carbides, intended for tools for processing wood-based materials, in conditions of technically dry friction. WC-Co composites were obtained using the innovative U-Fast technology. The coefficient of friction and wear intensity of the tribological system were assessed. The parameters were selected in such a way as to reflect the operating conditions of the tested materials as closely as possible.

## 2. Experimental Procedure

**Figure 1:**
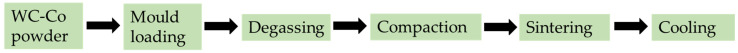
A diagram depicting the sintering process using the U-FAST method.

Samples obtained in a previous paper were used for this study [28] and sinters were prepared using a nanometric mixture of WC-5%wt.Co powder, with a WC grain size of 100 nm. Each time, an appropriate weight of powder was placed in a graphite matrix. The sample preparation process consisted of the following steps, shown in Figure 1.

The powders used for consolidation had irregular shapes and formed agglomerates of varying sizes. The WC-Co powders were sintered using a U-FAST (upgraded field-assisted sintering technology) machine. An essential aspect of U-FAST technology is that the sintering process can be conducted at significantly lower temperatures compared to other available methods such as pressureless sintering, hot isostatic pressing (HIP), and hot pressing (HP). As a result, this method reduces grain growth and significantly enhances the performance of the produced materials. FAST offers advantages such as a short sintering time, material homogeneity, and high energy efficiency through direct heat generation inside the material. The new U-FAST device effectively utilises all the benefits of FAST/SPS (spark plasma sintering) technology by employing short electric pulses at several tens of times higher voltage than typical SPS devices. The choice of temperature and holding time was experimental. Sintering was conducted at a temperature of 1220 °C with a holding time of 10 min. The process occurred under a load of 100 MPa. Finally, the samples were cooled in a vacuum of 10^−6^ mbar. After that, the sinters underwent a mechanical treatment, which involved bilateral surface polishing to reach a sample thickness of 2 mm. We measured the hardness using a Vickers hardness tester with a constant load of 30 kg. We took hardness measurements along the diameter of the sintered samples. In order to ascertain the fracture toughness (*K_IC_*), we measured the radial crack length around the Vickers indentation and applied Shetty’s formula. Table 1 presents a summary of the properties of the composites used for tribological testing.

**Figure 2:**
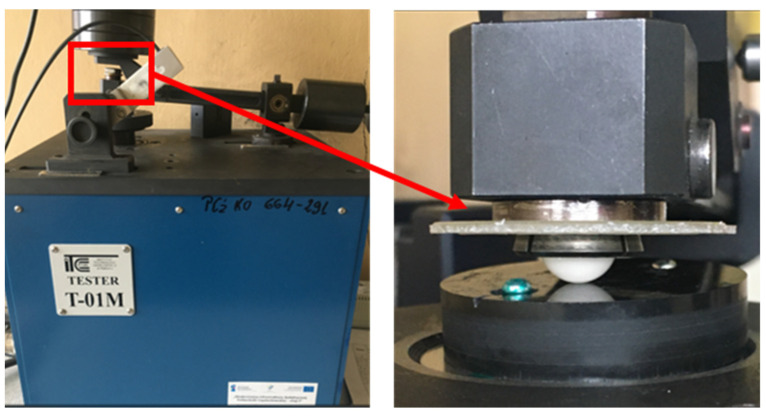
T-01 tribological tester and the Al_2_O_3_ ceramic ball-on-disc.

The tribological tests were carried out using a tribotester—T-01 method ball-on-disc (Figure 2). An Al_2_O_3_ ceramic ball with a diameter of 10 mm was used for the tests, and the tests were carried out under conditions of technically dry friction. The chemical composition of the ceramic balls used in the tests is presented in Table 2.

The following test parameters were used: pressing force of 55 N, total friction distance of 3000 m, rotational speed of 500 rpm, and radius of 3 mm. In order to determine the kinetics of abrasive wear, the total friction distance was divided into 15 sections of 200 m each. After each cycle, the samples were degreased with ethyl alcohol in an ultrasonic cleaner for 7 min, then dried and weighed using an OHAUS PX125D analytical balance with a measuring accuracy of five decimal places. The wear rates were calculated using the weight loss Δ*G* of the samples after the whole abrasion cycle. During the test, the friction force was recorded, enabling the determination of the friction coefficient. Before the test and after the 5th, the 10th and 15th cycles, using the Keyence VHX-7000 microscope, the surfaces of the samples were observed and surface roughness parameters were evaluated: Ra—arithmetical mean roughness value; Rt—total height of the roughness profile; and Rz—mean roughness depth. Three-dimensional surface images were also created. The obtained data enabled the characterisation of the wear process of the tested materials.

## 3. Results

**Figure 3:**
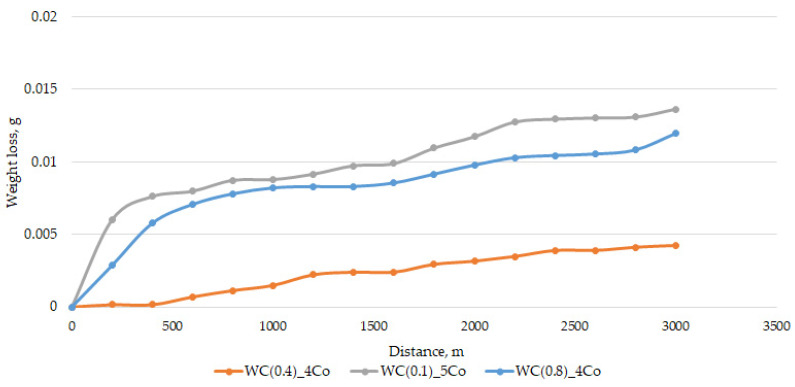
Change in weight of samples during successive friction cycles.

To establish the wear kinetics, Figure 3 depicts the weight loss variation in the tested WC-Co composites during successive friction cycles. During the initial stage of the process, the samples with the smallest WC grain size—0.1 µm—exhibited the most significant weight loss. Later on, the wear process was less intense, resulting in weight loss similar to that of composites with an average WC grain size of 0.8 µm. Among the samples with a submicron grain size, those containing 0.8 µm WC grains showed more intensive removal. Composites containing 0.4 µm WC grains showed the least weight loss.

**Figure 4:**
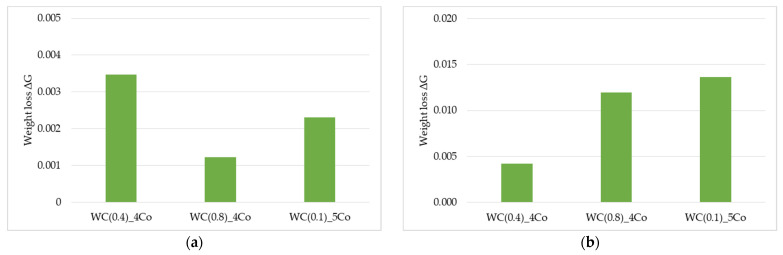
Weight loss ΔG after the whole abrasion cycle: (**a**) ball and (**b**) sample.

The mass component analysis of the friction node revealed that in submicron-sized grain composites (0.4 and 0.5 µm), lower sample mass consumption resulted in higher ball mass consumption, as shown in Figure 4. In contrast, nanometric WC-Co composites showed the highest sample mass consumption with simultaneous high ball mass loss. The sample that contains 0.4 µm WC grains exhibits a significantly lower weight loss value of ΔG. Figure 3 shows that the sample loses its weight steadily over the 3–4 cycles. Both samples containing a WC grain size of 0.1 µm and 0.8 µm exhibit two steps after cycles 3 and 8. This is a common phenomenon that results from the surface wear of the working layer and the occurrence of flaking off when the maximum strength of the joint is surpassed. This phenomenon occurs due to the micro-impacts that take place during frictional wear. Even minor irregularities can lead to an increase in the amplitude of vibrations, which raise local stresses and strains and eventually exceed the joint’s strength limit. Surprisingly, the best tribological resistance was not observed in the material with the smallest grain size. The wear observed in the bead used as a counterexample supports the results presented in Figure 3. The observed low bead wear for the 0.8 µm sample is noteworthy, as it indicates that this material has the poorest tribological properties under the tested conditions [40].

**Figure 5:**
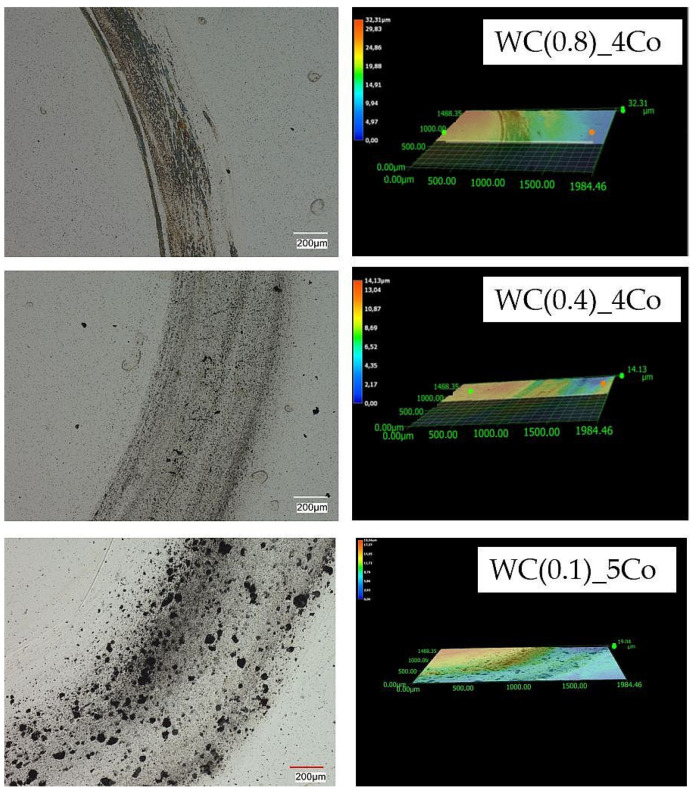
Topography of 2D and 3D wear marks on WC-Co composites surfaces after 5 friction cycles.

**Figure 6:**
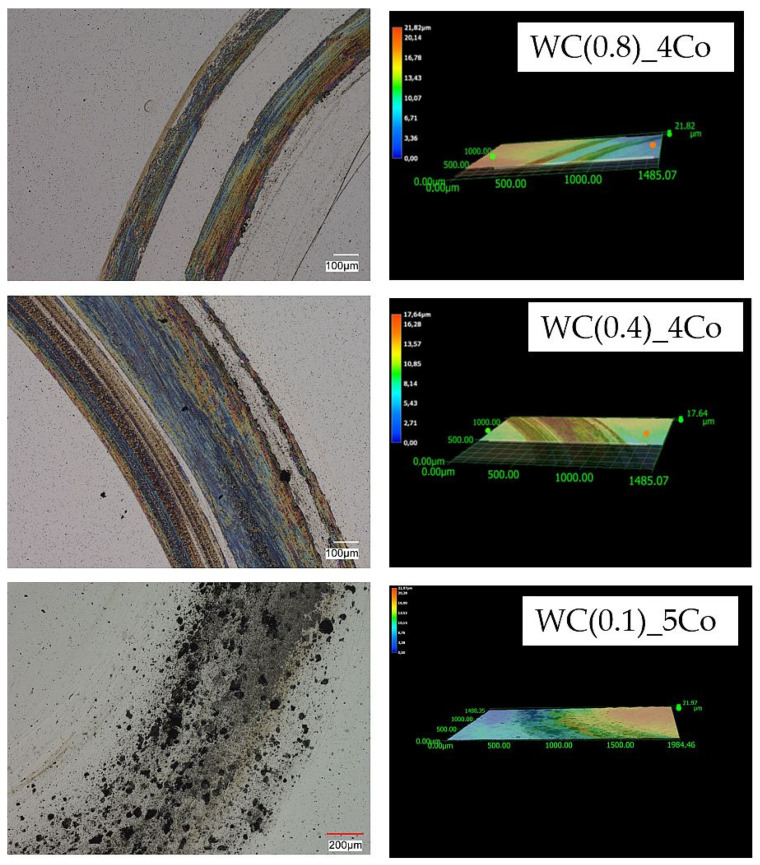
Topography of 2D and 3D wear marks on WC-Co composites surfaces after 10 friction cycles.

**Figure 7:**
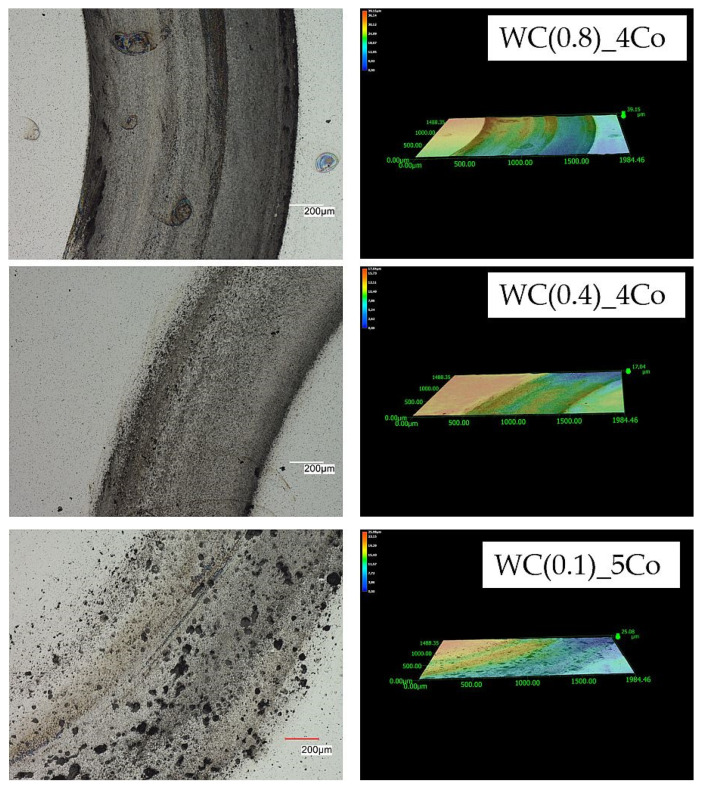
Topography of 2D and 3D wear marks on WC-Co composite surfaces after 15 friction cycles.

To investigate the mechanism of tribological wear for the tested sinters, we observed the sample surfaces after cycles of 5, 10, and 15 using a Keyence VHX-7000 microscope. After tribological testing, the surface texture and profiles of the samples are presented in Figure 5, Figure 6 and Figure 7. The obtained images reveal the effect of mechanical surface wear, caused by the collaboration of matrices in a friction node. Due to friction, the material undergoes micro-cutting, leading to intensified wear products within the mating surfaces, thus accelerating the surface degradation process. These observations revealed that abrasion was the predominant wear mechanism. The procedure led to an elevation in roughness, as verified by the measurements of the Ra, Rz, and Rt parameters. The figures display an evident distinction in the wear paths of the diverse materials. The wear track is hardly visible after 5 cycles for a sample with an average WC grain size of 0.4 µm (Figure 5). Materials obtained via powders with an average WC grain size of 0.8 µm and 0.1 µm have much greater profile depth. Previous studies [41,42,43] have shown that ultra-fine-grained carbides (with an average tungsten carbide size of 0.1–0.6 μm) possess higher hardness and wear resistance. However, it should be noted that they are also more susceptible to fracture compared to coarse-grained carbides, and this fact must be considered in the design of cutting tools. The microscopic images of wear shown in Figure 5, Figure 6 and Figure 7 also confirm the results described by the global factor of weight loss. Microscopic examination of the surface clearly shows uniform wear for the 0.4 µm sample. Very importantly, the 0.1 micron sample shows clear effects of bumps and chipped grains, indicating very uneven wear. Such a surface image indicates that this material will, by and large, be the worst-performing.

The analysis of the microstructure after 15 cycles, as shown in Figure 7, also confirmed a smaller loss of material due to friction in a sample made of a powder with an average grain size of WC 0.4 µm. The depth of the profile in the other tested materials is much greater. Observations of the surface topography and signs of wear of submicron samples clearly indicate the presence of a characteristic loss of material, caused by micro-cutting and the detachment of irregularities, which results from the interaction of hard wear products occurring between the rubbing surfaces. In the case of a nanometre sample, the surface is very rough and contains large cavities due to significant surface cracking, which indicates heavy wear of the material. First, cobalt is removed, which is a binding material for hard WC grains [44]. In place of cobalt clusters, visible craters are formed. Then, the WC carbide grains are degraded and pulled out.

**Figure 8:**
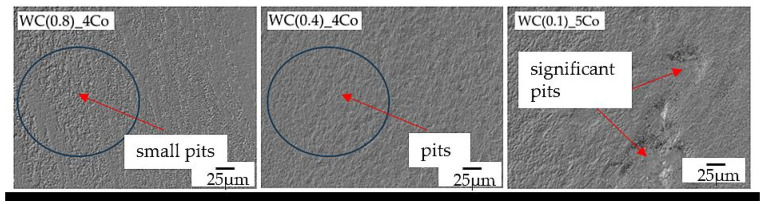
Worn surfaces of the tested WC-Co samples, Keyence VHX-7000 microscope, SEM technique, area 1000×.

Figure 8 shows the worn surface of the tested WC-Co composites. Observation of the surface reveals that the main features are the furrows seen in Figure 5, Figure 6 and Figure 7 and the pits (Figure 8). In particular, in samples with the smallest gradation of WC grain (0.1 µm), significant pits in the material are visible [45].

Confirmation of the analysis of microstructure images was obtained by examining the basic roughness parameters, as presented in Table 3.

**Figure 9:**
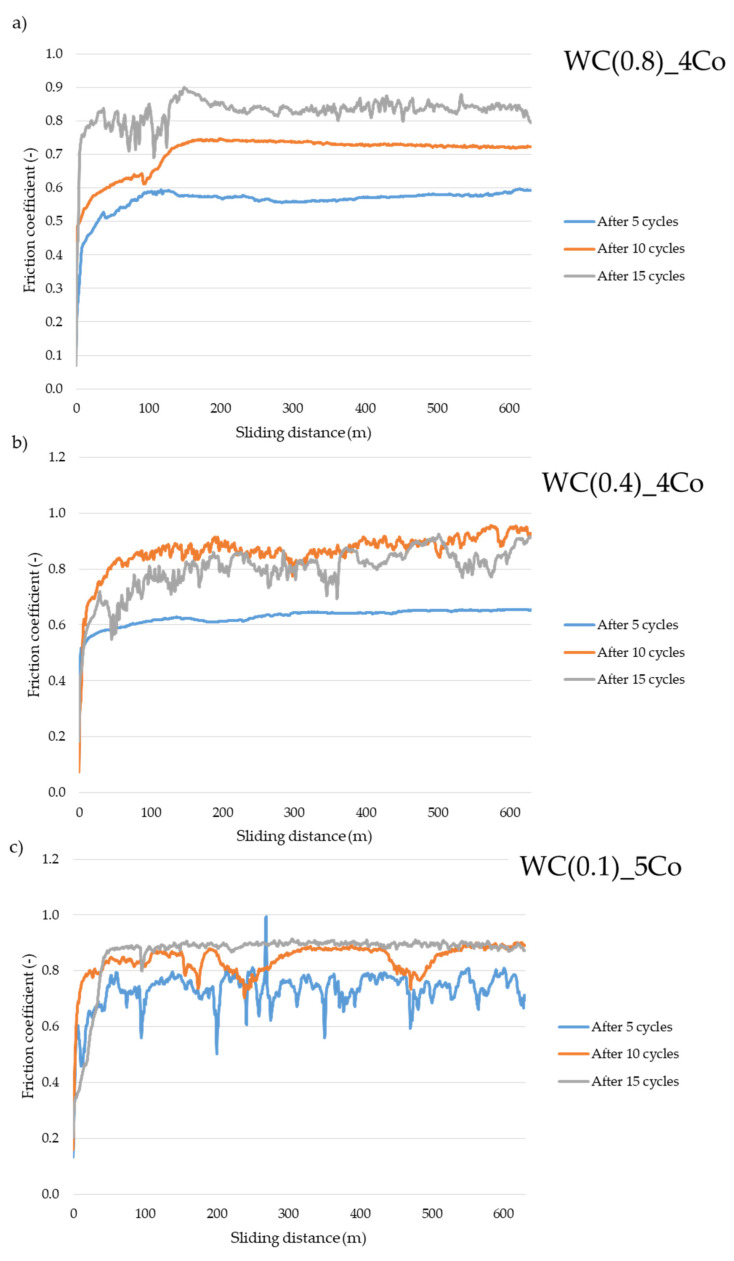
Values of the friction coefficient µ as a function of the friction path after 5, 10, and 15 cycles for WC-Co composites with the following average WC grain sizes: (**a**) 0.8 µm, (**b**) 0.4 µm, and (**c**) 0.1 µm.

Figure 9 shows the dependence of the friction coefficient as a function of distance, determined after 5, 10, and 15 cycles. For WC-Co composites, with an average WC grain size of 0.8 and 0.1 µm, the coefficient value increased with the number of cycles. However, for WC-Co composites, with a WC grain size of 0.4 µm, the highest friction coefficient value was obtained after 10 cycles. For submicron sintering, the average coefficient of friction, after 5 and 10 cycles, was relatively stable. However, for nanometric WC-Co, after 5 and 10 cycles the friction coefficient varied significantly over time. The significant dynamics of the changes in the friction coefficient values for the sinters with the finest WC grain may be due to the inhomogeneity of this material. It is extremely difficult to produce a homogeneous material using nanopowders, as they show a significant tendency to form agglomerates. These findings are also confirmed by the authors of [43], who studied the influence of WC grain size (the following WC grain sizes were tested: 0.27 μm, 0.47 μm and 0.75 μm) on the properties of WC-Co composites. No effect of WC particle size on the powder compaction process was observed. The average grain size of WC powder plays a significant role in the construction of the composite microstructure. The sintered materials obtained from the finest powder exhibit a heterogeneous microstructure containing significant cobalt clusters. In comparison with the other composites tested, they demonstrate significantly lower flexural strength values (0.28 μm—2200 MPa vs. 0.47 μm—3100 MPa).

## 4. Summary

In this preliminary study, samples of WC-Co composites, intended as blades for machining wood-based materials, were characterised from the tribological point of view. This paper presents the results of a comparative wear resistance study including wear trace measurements using a 3D profilometer and microstructure studies of submicron and nanostructured WC-Co carbides. The tests showed higher wear resistance for submicron carbides with an average grain size of WC-0.4 µm. In addition to having high abrasion resistance, the composites demonstrated a high hardness of 2270 HV30. In contrast, the fracture toughness coefficient was K_IC_ 8.33 MPa m^1/2^. Based on the test results, it was concluded that hardness alone cannot be the primary criterion for evaluating the abrasion wear resistance of WC-Co carbides because, as this study shows, material homogeneity has a significant effect on tribological resistance for nanometric WC grain size carbides. Although the 2192 HV30 hardness level was high, the nanocomposites with a WC grain size of 0.1 µm had lower abrasion wear resistance. In general, for all materials tested, the coefficient of friction increased with the number of cycles, i.e., as the contact area of the friction pair increased. Further studies of nanostructured carbides are planned to determine the reasons for the reduced resistance to abrasive wear.

## Figures and Tables

**Table 1:** Mechanical properties of the tested WC-Co samples.

Sample	WC Grain Size (µm)	Cobalt Content (% wt.)	Hardness (HV30)	*K_IC_* (MPa m^1/2^)	Technology	Literature
WC(0.4)_4Co	0.4	4	2270	8.33	U-FAST	[28]
WC(0.8)_4Co	0.8	4	2085	8.36	U-FAST	[28]
WC(0.1)_5Co	0.1	5	2192	9.27	U-FAST	This work

**Table 2:** Chemical composition of the Al_2_O_3_ ceramic balls examined in this study.

Compound (unit)	Contents
Al_2_O_3_ (%)	99.8
Na_2_O (ppm)	500
CaO (ppm)	200
MgO (ppm)	500
SiO_2_ (ppm)	350
Fe_2_O_3_ (ppm)	180

**Table 3:** Average values of selected surface roughness parameters obtained after successive cycles.

Material	Cycle Number	Roughness Parameters		
		Ra (µm)	Rt (µm)	Rz (µm)
WC(0.8)_4Co	5	2.23	11.42	11.44
	10	2.77	10.03	10.03
	15	5.24	18.96	18.97
WC(0.4)_4Co	5	1.30	7.90	7.90
	10	1.48	6.10	6.10
	15	1.58	5.84	5.84
WC(0.1)_5Co	5	2.46	12.61	12.61
	10	2.77	10.03	10.03
	15	5.24	18.96	18.96

## Data Availability

Not applicable.
